# The cost of hospital-acquired complications for older people with and without dementia; a retrospective cohort study

**DOI:** 10.1186/s12913-015-0743-1

**Published:** 2015-03-08

**Authors:** Kasia Bail, John Goss, Brian Draper, Helen Berry, Rosemary Karmel, Diane Gibson

**Affiliations:** Faculty of Health, University of Canberra, Canberra, Australia; Department of Old Age Psychiatry, University of New South Wales and Prince of Wales Hospital, Sydney, Australia; Data Linkage Unit, Australian Institute of Health and Welfare, Canberra, Australia

**Keywords:** Australia, Aged, Dementia, Adverse events, Nosocomial infection, Nursing, Length of stay, Cost

## Abstract

**Background:**

Increased length of stay and high rates of adverse clinical events in hospitalised patients with dementia is stimulating interest and debate about which costs may be associated and potentially avoided within this population.

**Methods:**

A retrospective cohort study was designed to identify and compare estimated costs for older people in relation to hospital-acquired complications and dementia. Australia’s most populous state provided a census sample of 426,276 discharged overnight public hospital episodes for patients aged 50+ in the 2006–07 financial year. Four common hospital-acquired complications (urinary tract infections, pressure areas, pneumonia, and delirium) were risk-adjusted at the episode level. Extra costs were attributed to patient length of stay above the average for each patient’s Diagnosis Related Group, with separate identification of fixed and variable costs (all in Australian dollars).

**Results:**

These four complications were found to be associated with 6.4% of the total estimated cost of hospital episodes for people over 50 (A$226million/A$3.5billion), and 24.7% of the estimated extra cost of above-average length of stay spent in hospital for older patients (A$226million/A$914million). Dementia patients were more likely than non-dementia patients to have complications (RR 2.5, *p* <0.001) and these complications comprised 22.0% of the extra costs (A$49million/A$226million), despite only accounting for 10.4% of the hospital episodes (44,488/426,276). For both dementia and non-dementia patients, the complications were associated with an eightfold increase in length of stay (813%, or 3.6 days/0.4 days) and doubled the increased estimated mean episode cost (199%, or A$16,403/A$8,240).

**Conclusion:**

Urinary tract infections, pressure areas, pneumonia and delirium are potentially preventable hospital-acquired complications. This study shows that they produce a burdensome financial cost and reveals that they are very important in understanding length of stay and costs in older and complex patients. Once a complication occurs, the cost is similar for people with and without dementia. However, they occur more often among dementia patients. Advances in models of care, nurse skill-mix and healthy work environments show promise in prevention of these complications for dementia and non-dementia patients.

## Background

Common hospital-acquired complications that occur in Australian hospitals, such as urinary and respiratory infections, cost more than expensive but uncommon events such as surgical misadventure and drug-resistant infections [1]. Though people aged over 65 use nearly one-half (48%) of hospital bed days [2], there are few studies of hospital-acquired complications in this population [3]. The costs associated with this group are likely to be substantial as patients over 70 years have higher rates of mental state alterations, such as delirium, and urinary and respiratory tract infections [4].

Complications among older people are often assumed to result from being old and are therefore seen as unavoidable [5]. However, a recent systematic review found disproportionately more hospital-acquired complications in older than in younger patients – accounted for by complexity, frailty and comorbidity rather than by age itself [3]. These complications may thus be preventable, potentially offering large cost savings. Indeed, rates of delirium, urinary tract infections and pneumonia among older patients are modifiable with particular models of care, such as dedicated geriatric orthopaedic wards [6], and multidisciplinary, integrated and non-pharmacological approaches to inpatient management [7]. Nursing circumstances, such as lower proportions of registered nurses [8], increased nurse overtime and working hours [9] and elevated nurse manager turnover [10] are also associated with increased rates of these complications. Nurses working in such environments may not be able to offer adequate care to older patients who tend to experience complexity, frailty and comorbidity and, therefore, need more attention.

An important example of complexity and comorbidity that is common among older patients is dementia. People with dementia cost on average A$2,710 more per hospital episode than do those without dementia [11]. They also have significantly higher rates of hospital-acquired complications which are known to be sensitive to nursing care, including urinary tract infections, pressure ulcers, pneumonia and delirium [12] some of which, we argue, may be preventable.

We aimed to estimate the cost of older patients discharged from public hospitals in the Australian state of New South Wales (NSW) with one of four nurse-sensitive hospital-acquired complications (urinary tract infections, pneumonia, pressure ulcers or delirium) and to compare costs for people with and without dementia.

## Method

### Data source and coding

The study was nested in the Australian Hospital Dementia Services Project [13-15] which uses hospital discharge data from the 2006–2007 financial year (July to June) for all public hospital overnight discharges for episodes of care for people aged 50 and over (50+) in the Australian state of New South Wales (NSW) (‘study data’). NSW is Australia's most populous state with a diverse population from metropolitan to remote areas and a range of hospital-based and community-based dementia services. Ethics approval was obtained from the NSW Population and Health Services Research Ethics Committee. Using a person identifier, patients were coded as having dementia if dementia was ever documented as a principal or additional diagnosis (AR-DRG ICD10 codes include F00, F01, F02, G30, G31) in any hospital stay over a two-year period, offering a high capture rate and minimising selection bias [14].

Internationally valid patient-level and risk-adjusted coding rules for adverse outcomes have identified a number of nurse-sensitive hospital-acquired complications [8,12,16]: urinary tract infection, pressure ulcer, pneumonia, deep vein thrombosis/pulmonary embolism, gastrointestinal bleeding/ulcer, central nervous system complications (e.g., delirium), shock/cardiac arrest, surgical wound infection, pulmonary failure, physiological/metabolic failure (e.g., electrolyte imbalance) and failure to rescue (death following sepsis, pneumonia, gastrointenstinal bleeding or shock).The coding rules are conservative, excluding patients at risk of developing complications due to their underlying aetiology, so that complications identified using these coding rules are likely to result from hospitalisation. For example, patients who have paralysis (G80 and G84); skin conditions (Major Diagnostic Category 9) as a primary or secondary diagnosis; pressure ulcer as a secondary diagnosis (L89); or length of stay more than 4 days are excluded from the complication ‘pressure ulcer’ (L89). For all complications, episodes with length of stay (LOS) beyond 90 days were excluded to maintain consistency of the approach to these complications with other studies [16]; this approach excluded 1,268 episodes, or 0.3% of the study population. Detailed description of the method for deriving nurse-sensitive outcomes from hospital discharge data is available in other publications [8,16,17]. Recent research [12] has highlighted that people with dementia have higher rates of four of the nurse-sensitive hospital-acquired complications compared to people without dementia (urinary tract infections with dementia 13.4% compared to 7.9% without dementia, pneumonia 4.8% compared to 3.5%, pressure ulcers 5.9% compared to 3.8% or delirium 4.0% compared to 1.5% respectively); consequently these were the four complications examined for this study.

### Calculating the cost of length of stay

Publically available hospital data were used to calculate average LOS for each Diagnosis Related Group (DRG) for NSW for the financial year July 2006 to June 2007 [18]. Each DRG represents a class of patients with similar clinical conditions requiring similar hospital services. The average LOS for each DRG usually includes day-stay patients but, for this analysis, these short stays were excluded so that we could compare LOS with the study’s overnight population. Publically available hospital data were also sourced for the total average cost of patient discharges by DRG by state for the financial year 2006–07. NSW public hospitals provide estimates of costs by DRG broken down into treatment expense subcategories by age-group [19]. We refer to the DRG, LOS and costs data as ‘state data’ (which included all patients from ages 0+, whereas our study data only included those aged 50+).

We used an established method, utilising the cost subcategories, to calculate which costs are dependent on LOS [20]. These estimated costs can be ‘variable’ (ward nursing, ward medical, non-clinical salaries, pathology, imaging, allied health, pharmacy, supplies, on-costs, hotel, depreciation) or one-off ‘fixed’ (critical care, operating rooms, emergency departments, special procedure suites, prosthesis). Estimated variable and fixed costs were calculated separately for each DRG and each LOS (Table 1), so that costs for each of the 426,276 hospital episodes in the study were estimated.

**Table 1:** **Calculations to estimate cost** ^^^

**Patient DRG**	**Data source**	**Fractured neck of femur, +c/c**
Fixed costs (estimated)	State data	$670
Variable costs (estimated)	State data	$6,570
Average length of stay for the DRG	State data	8 days
Daily variable cost	State data	$6,570/8 = $820
Actual length of stay	Study data	11 days
Actual estimated cost	Study data * State data	11 * $820 + $670 = $9690
***If patient length of stay is above the average for the DRG:***		
*Extra length of stay*	*Study data – State data*	*11 - 8 = 3 days*
*Extra cost*	Study data * State data	3 * $820 = $2460

^^^Example uses Fractured Neck of Femur with complication and/or comorbidity (+c/c) (DRG I78A), figures rounded to reflect estimation status.

DRG = Diagnostic Related Group.

State data = all ages overnight NSW public hospital episodes.

Study data = 50+ overnight NSW public hospital episodes.

**Figure 1:**
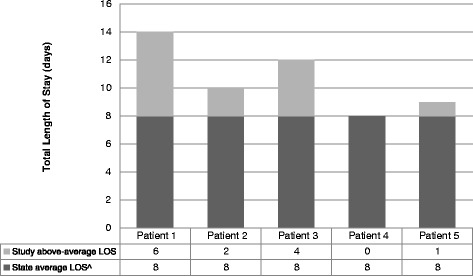
**Illustration of how above-average length of stay (LOS) was determined.** Using example DRG I78A, which has a state average LOS of 8 days. (DRG = Diagnosis Related Group).

Patients with above-average LOS are of interest because the LOS is a modifiable component which may be responsive to interventions. Patients who stayed longer than the all-ages overnight state average for their DRG were considered to have ‘above-average’ LOS. The state-average LOS was subtracted from these patients’ LOS to calculate the number of additional days that each ‘above-average’ patient stayed (Figure 1). To calculate the estimated ‘extra costs’ for these patients, we multiplied their additional days by the daily *variable* cost for their DRG (i.e., excluding the one-off fixed costs that do not change with LOS) (see section with italicised text, Table 1).

### Analytic approach

The Charlson index was derived from the AR-DRG from each hospital episode to provide a summary measure of patient comorbidity [21]. The Charlson index assigns a weighted score to each of 17 comorbidities (diabetes, hemiplegia or paraplegia, any cancer, HIV/AIDS and major cardiovascular, renal, rheumatic, peptic ulcer and liver diseases), based on the relative risk of 1-year mortality. The sum of the Charlson index score is used to indicate disease burden. Mean cost weights were derived from the state data, and are an average score of complexity for patient episodes by comparing the average cost of a DRG to the average cost of all DRGs. The mean cost weight was applied to the episode DRG in the study data as a reflection of patient acuity. Pearson’s χ^2^ test of independence was used to test the magnitude of association and goodness-of-fit of the relative risk of complications comparing dementia and non-dementia groups. All analyses were conducted using SAS EG V.9.2 with records with missing data excluded from analysis as required. Missing data were rare in the variables used in this analysis. Diagnosis information was missing in less than 0.2% and sex for less than 0.001% of records for 2006–07; DRG data were always present. As the data are a census sample, and therefore contain the complete population, age-standardisation has not been applied.

## Results

There were 426,276 overnight hospital episodes for patients aged 50+ in the financial year 2006–07 (215,455 female, 44,488 with dementia). The largest age group was 75–84 years which had 130,127 episodes. For people 50+ *without* dementia, the average length of stay (LOS) was 7.1 days, 0.3 days above the all-ages average. For people 50+ *with* dementia, the average LOS was 10.9 days, 1.6 days more than the all-ages state average and 3.8 days more than for non-dementia patients. The total estimated cost for hospital care for people 50+ in NSW was A$3,512 m (Table 2), equivalent to US$5,010 m (converted using ‘purchasing power parity’ for 2006 and 2007 ratios from www.imf.org). Of the variable costs, ward nursing accounted for 34% and ward medical 18%. Operating rooms accounted for 48% and critical care 26% of the fixed costs.

**Table 2:** **Characteristics and estimated costs of public hospital discharge episodes ages 50+, NSW 2006-7**

		**Complication acquired**			**No complication acquired**			**Whole Sample**		
		**Dementia**	**No dementia**	**Total**	**Dementia**	**No dementia**	**Total**	**Dementia**	**No dementia**	**Total**
**Characteristics**	Group Rate (hospital episodes)^#^	9,751	33,501	43,252	34,737	348,287	383,024	44,488	381,788	426,276
	Mean Charlson Score^#^	1	1.6	1.5	0.9	1	1	0.9	1	1
	Mean Cost Weight^#^^	2.7	3.5	3.3	1.9	1.7	1.7	2	1.9	1.9
**Means**	Mean Study LOS (days)^#^	15.4	15.4	15.4	9.7	6.3	6.6	10.9	7.1	7.5
	Mean State LOS (days)^^^	11.4	11.8	11.8	8.7	6.3	6.5	9.3	6.8	7.1
	Mean Extra LOS (days)^#^^	4	3.5	3.6	0.9	0	0.1	1.6	0.3	0.4
	Mean Cost (A$)^#^^	$14,058	$17,086	$16,403	$8,661	$7,185	$7,319	$9,844	$8,054	$8,240
**Totals**	Total LOS (days)^#^	150,095	515,669	665,764	335,663	2,203,419	2,539,082	485,758	2,719,088	3,204,846
	Total Cost (A$)^#^^	$137,082,050	$572,399,374	$709,481,424	$300,844,462	$2,502,329,287	$2,803,173,749	$437,926,512	$3,074,728,661	$3,512,655,173

^#^Study data ^^^State data ^#^^Derived from State and Study data A$ All costs in Australian Dollars (2007).

We found that dementia patients were over-represented in key complications. In NSW public hospitals, among patients aged 50+, 10.4% (44,488/426,276 episodes) had documented dementia and 10.1% (43,252/426,276) experienced at least one of the key hospital-acquired complications. Yet 22.5% (9,751/43,252) of the episodes with complications were for dementia patients; and 28.8% of multiple complications (1,362/4,728 episodes, data not shown) were for dementia patients. While 21.9% of dementia patients (9,751/44,488 (*p* < 0.001)) suffered a complication, only 8.8% of non-dementia patients did so (33,501/381,788 (*p* < 0.001)), giving dementia patients a 2.5 relative risk of acquiring a complication.

A similar pattern was found for use of hospital bed days. Dementia patients were only 10.4% of the sample but they accounted for 15.2% (485,758/3,204,846 days) of total hospital bed days and 22.5% (150,095/665,764 days) of the total bed days for episodes that included an acquired complication. In examining costs, episodes with a complication accounted for 31.3% of dementia patient total estimated costs (A$137 m/$438 m) compared to 18.6% of non-dementia costs (A$572 m/A$3,075 m).

Episodes with above-average LOS are of interest as additional days spent in hospital are a modifiable component for cost savings, and 51.6% (22,309/43,252) of patient episodes with a complication had an above-average LOS (Tables 2 and 3). Looking only at the 50+ population with above-average LOS, complications were associated with 24.7% of the estimated cost of additional days spent in hospital in 2006–07 in NSW (A$225 m/A$914 m) (Table 3). That is, these four complications accounted for one-quarter of the additional costs of patients with above-average LOS.

**Table 3:** **Characteristics and estimated costs of public hospital discharge episodes with above-average length of stay**

		**Complication acquired**			**No complication acquired**			**Whole sample**		
		**Dementia**	**No dementia**	**Total**	**Dementia**	**No dementia**	**Total**	**Dementia**	**No dementia**	**Total**
**Characteristics**	Group Rate (hospital episodes)^#^	5,176	17,133	22,309	13,584	121,285	134,869	18,760	138,418	157,178
	Mean Charlson Score^#^	1.1	1.7	1.6	0.9	1.1	1.1	1	1.2	1.1
	Mean Cost Weight^#^^	2.5	3.3	3.1	1.8	1.6	1.6	2	1.8	1.9
**Means**	Mean Study LOS (days)^#^	23.1	23.5	23.4	18.2	12.1	12.7	19.5	13.5	14.2
	Mean State LOS (days)^^^	10.9	11.5	11.3	8.4	6.3	6.5	9.1	6.9	7.2
	Mean Extra LOS (days)^#^^	12.2	12	12	9.7	5.8	6.2	10.4	6.6	7
	Mean Cost (A$)^#^^	$19,873	$23,803	$22,891	$15,154	$11,793	$12,132	$16,457	$13,280	$13,659
**Totals**	Total LOS (days)^#^	119,512	401,844	521,356	247,096	1,469,720	1,716,816	366,608	1,871,564	2,238,172
	Total Cost (A$)^#^^	$102,863,592	$407,811,537	$510,675,130	$205,861,352	$1,430,365,193	$1,636,226,545	$308,724,944	$1,838,176,730	$2,146,901,675
**Above-average length of stay**	Total Above-Average LOS (days)^#^^	63,277	204,755	268,032	132,327	707,118	839,444	195,604	911,873	1,107,476
	Mean Episode Cost of Above-Average LOS (A$)^#^^	$9,580	$10,284	$10,120	$7,428	$4,843	$5,103	$8,022	$5,516	$5,875
	Total Cost of Above-Average LOS (A$)^#^^	$49,585,461	$176,188,003	$225,773,464	$100,903,070	$587,382,179	$688,285,249	$150,488,531	$763,570,182	$914,058,713

^#^Study data ^^^State data ^#^^Derived from State and Study data A$ All costs in Australian Dollars (2007).

Above-Average LOS = Length of stay longer than the all-ages state average for the patient's DRG, LOS = length of stay.

**Figure 2:**
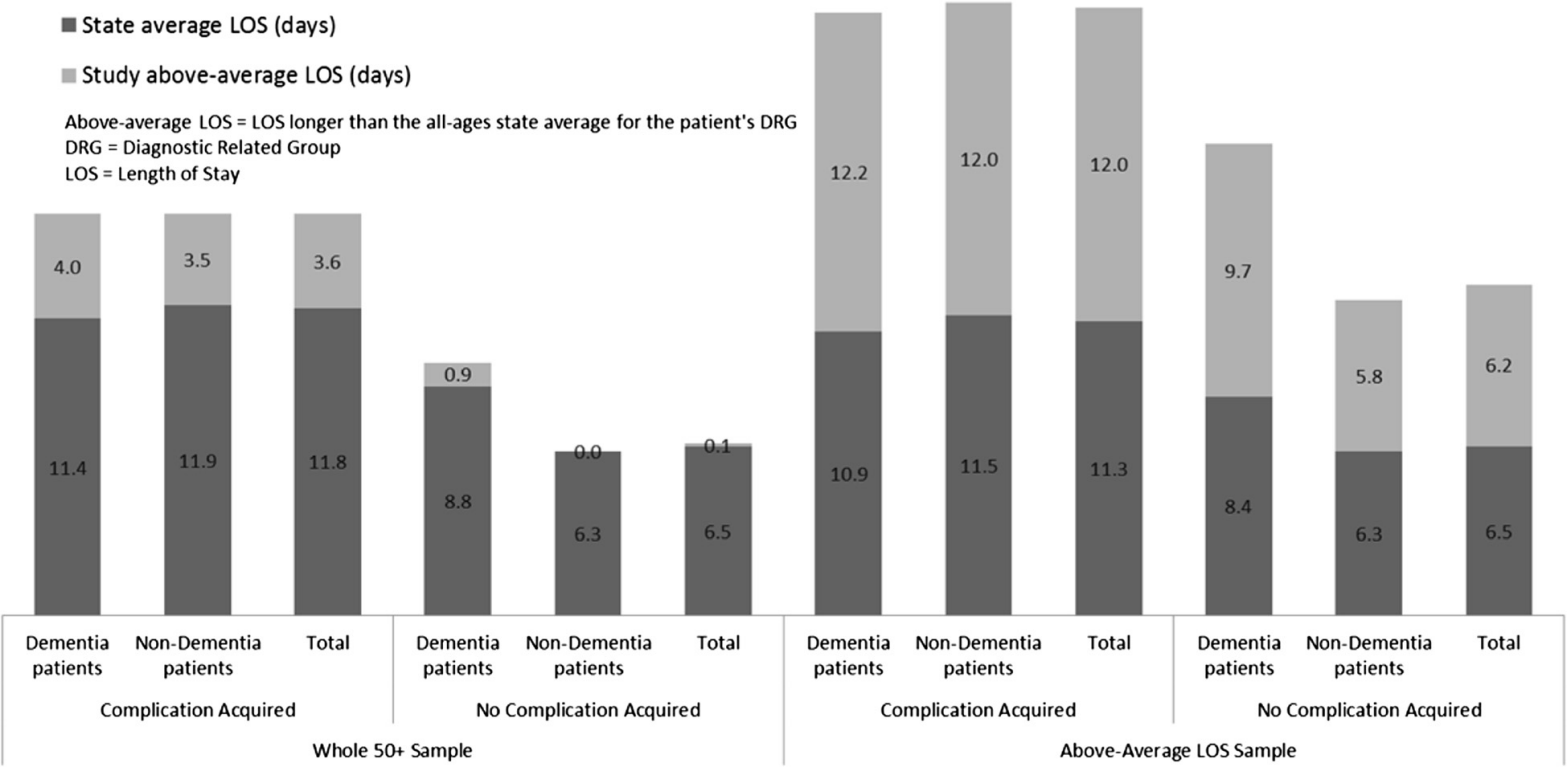
**Comparison of samples, comparing dementia and complication status.**

In the whole 50+ population, episodes with a complication averaged 3.6 days above-average LOS and cost A$16,403 (Table 2 and Figure 2). Episodes with a complication for patients with above-average LOS had 12 additional days and cost an estimated A$22,891, of which A$10,120 was associated with the above-average LOS (Table 3 and Figure 2). That totalled 5.8 days longer than people with above-average LOS without a complication. Comorbidity and complexity of patient episodes with complications was similar for the whole 50+ population and above-average LOS sample (Charlson index 1.6 to 1.5 and cost weight 3.1 to 3.3 respectively), indicating these variables had minimal effect on the above-average LOS.

The total estimated costs of above-average LOS for all patients 50+ with complications was A$226 m, with dementia patients accounting for 22.0% of these even though they were only 10.4% of the sample (Tables 2 and 3). At A$9,580, the mean cost of the above-average LOS for those with complications was slightly cheaper for dementia patients than for non-dementia patients (A$10,284) (Figure 2 and Table 3). Complications are thus associated with doubling the estimated cost of extra days for non-dementia patients and add 26% for dementia patients.

## Discussion

The costs associated with hospital-acquired complications in conjunction with dementia have not previously been quantified. In this study we examined the association between nurse-sensitive hospital-acquired outcomes and LOS to quantify the cost of four key complications (urinary tract infections, pressure ulcers, pneumonia and delirium) in people aged 50+ with and without dementia. Our findings indicate that people with dementia have more than double the rates of complications than people without dementia and, consequently, a disproportionately large amount of the total additional costs, even though their mean additional cost is actually slightly lower than that for people without dementia.

These findings from NSW reinforce and extend previous international findings on LOS and estimated costs, though comparisons of length of stay and costs are challenging given differences in health care systems. The increased LOS of 3.6 days attributable to the four key complications is similar to other research, ranging from 1 to 9 days [22]. Complications are more common in older adult inpatients and, though no more costly, they occur more often [4]. Similarly, we found that, once a complication occurs, the cost is similar for people with and without dementia but they occur more often among dementia patients. Most research on hospital-acquired complications in the elderly has focussed on physician-related adverse events with smaller sample sizes using case-note review [23,24] or has not included all four common complications studied here. However, a recent prospective study found that 30% of 60+ admissions resulting in an adverse event had dementia [24], not inconsistent with our finding that 22% of episodes aged 50+ resulted in one of four hospital-acquired complications among people with dementia.

Surprisingly, but consistent with previous research, complications made dementia patients’ *cheaper* than non-dementia patients. **With** complications, we found dementia patients cost an estimated A$3,028 per episode *less* than that for non-dementia patients (or A$704 for the above-average LOS sample), while episodes for dementia patients **without** complications cost A$1,476 *more* than non-dementia patients (or A$2,585 for the above-average LOS sample). Using a slightly different DRG classification, an examination of state-wide dementia costs focussing on ‘reason for hospital care’ [11] found that episodes for dementia patients **with ‘**complications and/or comorbidities’ cost A$389 *less* than episodes for non-dementia patients, while episodes for dementia patients **without** ‘complications and/or comorbidities’ cost A$2,729 *more* than non-dementia patients. This trend suggests that complications are vital in considering LOS and cost differences between dementia and non-dementia patients.

The heterogeneity of the older population has been cited as reason for lack of predictors and risk profile for elderly hospital patients acquiring complications [24]. However, several studies including dementia patients in their samples have demonstrated lower complication rates when certain models of care are used. For example, a comprehensive multidisciplinary program for hip fractures reduced confusion, pneumonia, urinary tract infection and LOS among all patients [6]. Elsewhere, when local clinicians initiated a multidisciplinary model of evidence-informed care, delirium decreased from 35% to 19% [7]. A meta-analysis of 14 trials revealed that an ‘Acute Care of the Elderly’ approach, including medical review, early rehabilitation and patient-centred care, was optimal in reducing delirium and pressure ulcers [25]. These fields of evidence demonstrate a paradigm shift acknowledging prevention of complications in complex inpatients as achievable and appropriate “as new scientific evidence of causal factors emerges, together with new research on effective prevention” [1] (p142).

A significant component of any of these care models is the quality and quantity of nurse staffing. Though no studies have focussed on older patients, higher proportions of registered nurses and lower workloads have been associated with decreased levels of the key complications [12]. However, it has previously been identified that reduced LOS alone may be insufficient financial incentive for hospitals to invest in increasing registered nurse staffing [22]. In our study, ward nursing accounted for over one-third of patient costs for the older in-patient population, but it remains unclear whether the DRG or case-mix funding systems appropriately estimate nursing care needs of patients more likely to get complications – that is, patients with dementia [26,27]. Further research could focus on administrative data and the accuracy of cost allocations via DRG for variable costs such as nursing, and may be able to explore workplace variations and efficiencies in hospital work environments that contribute to the hospital estimations of DRG costs. Cost estimations of these complications at the patient level would also be complementary, but difficult to achieve. Inclusion of episodes with ‘outlier’ length of stay (in this study, beyond 90 days) will be important in understanding the full impact of complex patients on hospital costs; and particularly gaining understanding about the kinds of complexity that make up complex patients. Multivariate analysis including key variables such as acuity, chronic comorbidity, and care dependency are also needed to contribute to international discussion on human and financial costs.

Study limitations include a reliance on hospital discharge data, though we are fortunate that Australia’s hospital data reliability and quality is highly regarded since the worldwide initiation of data dictionaries in the early 1990s (see [28]), and the study is strengthened by a comprehensive approach to data linkage modelled by the Australian Institute of Health and Welfare. Other limitations are the lack of ‘condition-onset flags’ [4] to improve accuracy in identifying hospital-acquired complications. However, our patient-level risk-adjustment approach is the most refined method currently published with this level of costing detail for the aged population. Other limitations of the study include the accuracy of medical record coding for dementia; we have ameliorated this to some extent with the 2-year look-back for any diagnosis of dementia in any public hospital. Our costing methods are limited in that costs are estimated only through association with DRG and LOS, and are compared to state average LOS for all ages, rather than reflecting our sample of 50+. However, by only including those episodes with an above-average LOS the costs estimated by this study are conservative.

## Conclusion

We found that four potentially preventable complications were associated with 6.4% of the total estimated cost of hospital episodes for people over 50 (A$226million/A$3.5billion), and 24.7% of the estimated extra cost of above-average length of stay spent in hospital for older patients (A$226million/A$914million). These complications are associated with an increased length of stay of 3.6 days and a mean episode cost of A$16,000 per patient. Dementia patients are 2.5 times more likely to experience complications than are non-dementia patients. As a consequence, people with dementia account for nearly one-quarter of the costs of above-average LOS, despite accounting for only one-tenth of hospital episodes for people aged 50+. These findings highlight that complications are key in examining and understanding the costs of length of stay, older patients, and dementia in hospital. Duty of care to patients and to taxpayers requires that the risks of acquiring complications while in hospital be mitigated. It is not clear how responsibility for prevention and mitigation is best shared between hospitals and the state, but research on acute models of aged care, nurse staffing, skill mix and healthy multidisciplinary work environments have shown promise in prevention of these key hospital-acquired complications.

## Ethics

Obtained from the NSW Population and Health Services Research Ethics Committee (HREC/08/CIPHS/49 and 2008/11/109), the Australian Institute of Health and Welfare Ethics Committee, the University of NSW and the University of Canberra Human Research Ethics Committees (08–85).

## Availability of supporting data

Statistical code and details are available from the corresponding author. Dataset inquiries can be made to the Australian Institute of Health and Welfare via the corresponding author.

## Abbreviations

LOS: Length of stay
DRG: Diagnostic related groups
DCRC: Dementia Collaborative Research Centres
NSW: New South Wales
